# The Expression of Transcription Factors Mecp2 and CREB Is Modulated in Inflammatory Pelvic Pain

**DOI:** 10.3389/fnsys.2018.00069

**Published:** 2019-01-11

**Authors:** Alison Xiaoqiao Xie, Xiao-Qing Pan, Randall B. Meacham, Anna P. Malykhina

**Affiliations:** ^1^Division of Urology, Department of Surgery, School of Medicine, University of Colorado Denver, Aurora, CO, United States; ^2^Division of Urology, Department of Surgery, University of Pennsylvania, Philadelphia, PA, United States

**Keywords:** inflammation, visceral pain, TNBS, Mecp2, CREB, dorsal root ganglia, spinal cord

## Abstract

Early activation of transcription factors is one of the epigenetic mechanisms contributing to the induction and maintenance of chronic pain states. Previous studies identified the changes in a number of nociception-related genes, such as calcitonin gene-related peptide (CGRP), substance P (SP), and brain-derived neurotropic factor (BDNF) in the pelvic organs after transient colonic inflammation. The gene and protein expression of these neuropeptides could be modulated by transcription factors Methyl-CpG-binding protein 2 (Mecp2) and cAMP response element-binding protein (CREB). In this study, we aimed to evaluate time-dependent changes in the expression levels of Mecp2 and CREB in the lumbosacral (LS) spinal cord and sensory ganglia after inflammation-induced pelvic pain in rat. Adult Sprague-Dawley rats were treated with 2,4,6-trinitrobenzenesulfonic acid (TNBS) to induce transient colonic inflammation. LS (L6-S2) spinal cord segments and respective dorsal root ganglias (DRGs) were isolated from control and experimental animals at 1, 2, 6, 24 h and 3 days post-TNBS treatment. Immunohistochemical (IHC) labeling and Western blotting experiments were performed to assess the expression of Mecp2, CREB and their phosphorylated forms. Total Mecp2 expression, but not phosphorylated p-Mecp2 (pS421Mecp2) expression was detected in the cells of the spinal dorsal horn under control conditions. Colonic inflammation triggered a significant decrease in the number of Mecp2-expressing neurons in parallel with elevated numbers of pS421Mecp2-expressing cells at 2 h and 6 h post-TNBS. The majority of Mecp2-positive cells (80 ± 6%) co-expressed CREB. TNBS treatment caused a transient up-regulation of CREB-expressing cells at 1 h post-TNBS only. The number of cells expressing phosphorylated CREB (pS133CREB) did not change at 1 h and 2 h post-TNBS, but was down-regulated by three folds at 6 h post-TNBS. Analysis of DRG sections revealed that the number of Mecp2-positive neurons was up-regulated by TNBS treatment, reaching three-fold increase at 2 h post-TNBS, and eight-fold increase at 6 h post-TNBS (*p* ≤ 0.05 to control). These data showed early changes in Mecp2 and CREB expression in the dorsal horn of the spinal cord and sensory ganglia after colonic inflammation, suggesting a possible contribution Mecp2 and CREB signaling in the development of visceral hyperalgesia and pelvic pain following peripheral inflammation.

## Introduction

The induction and persistence of chronic pain states involve epigenetic regulation of several nociception-related genes expressed in the dorsal horn neurons of the spinal cord and peripheral sensory ganglia (Geranton et al., 2008; Díaz de León-Guerrero et al., 2011; Geranton, 2012; Tochiki et al., 2012). Coordination of gene expression can be achieved through DNA CpG methylation which induces chromatin remodeling and modulation of gene activity through transcriptional complexes such as the Methyl-CpG-binding protein 2 (Mecp2). Mecp2 is a transcription factor that functions mostly as a gene repressor by binding to methylated DNA at methyl-CpG sites (Nan et al., 1998; Fuks et al., 2003). It is predominantly expressed in mature neurons, and mutations in *MECP2* gene are associated with Rett syndrome, a neurodevelopmental disorder characterized by severe deficit in the nervous systems (Amir et al., 1999) and altered pain sensitivity (Downs et al., 2010). Increased Mecp2 expression was identified in several animal models of pain including neuropathic pain (Manners et al., 2015, 2016), somatic pain (Hou et al., 2015), cutaneous pain (Tochiki et al., 2012), and inflammation induced hyperalgesia (Suzuki et al., 2016).

Prior functional and molecular studies from our laboratory identified an up-regulation of expression of calcitonin gene related peptide (CGRP), substance P (SP), and brain-derived neurotrophic factor (BDNF) genes in sensory and spinal neurons followed by subsequent release of these neuropeptides in the urinary bladder after transient colonic inflammation (Pan et al., 2010; Lei and Malykhina, 2012). It has been shown that neuronal depolarization could lead to calcium-dependent phosphorylation and release of Mecp2 from the promoter region of BDNF (Chen et al., 2003). Therefore, we focused on characterizing early changes in Mecp2 expression and phosphorylation which occurred within hours after colonic inflammation.

In the spinal cord, activation of Mecp2 triggered analgesic effects in acute pain model through regulation of cAMP response element-binding protein (CREB)/miR-132 pathway (Zhang et al., 2015). Both transcription factors, Mecp2 and CREB, were shown to be involved in neuronal plasticity in post-mitotic neurons (Carlezon et al., 2005; Fasolino and Zhou, 2017). Activation of CREB in sensory ganglia was observed as an early response to noxious stimulation prior to long-lasting peripheral sensitization (Freeland et al., 2000; Carlezon et al., 2005). Furthermore, in the absence of methylated CpG in the promoter region of BDNF gene, Mecp2 was able to activate BDNF transcription by binding unmethylated promoters and recruiting co-activators and CREB (KhorshidAhmad et al., 2016). CREB1 was also shown to be a direct Mecp2 target in hypothalamic neurons, and activation of Mecp2 increased expression of CREB1 gene *in vivo* (Chahrour et al., 2008). Therefore, in this study, we characterized the expression and phosphorylation of the transcription factors Mecp2 and CREB in the lumbosacral (LS) dorsal horn of the spinal cord and dorsal root ganglias (DRGs) after induction of transient colonic inflammation followed by long-term hyperalgesia and visceral pain.

## Materials and Methods

### Animals and Experimental Groups

Adult Sprague-Dawley rats (*N* = 60, Charles River Laboratories, 200–250 g) were used in this study. Rats were housed two per cage, with free access to food and water and maintained on a 12-h light/dark cycle. The studies were performed in accordance with institutional and national guidelines and regulations. The University of Colorado Denver Institutional Animal Care and Use Committee approved all experimental protocols involving animal use. Animal care was under the supervision of the University of Colorado’s Office of Animal Care Management in accordance with the Association for Assessment and Accreditation of Laboratory Animal Care (AAALAC) and National Institutes of Health guidelines. All efforts were made to minimize the potential for animal pain, stress or distress. The animals were divided into several control and experimental groups. Experimental animals underwent intracolonic 2,4,6-trinitrobenzenesulfonic acid (TNBS) treatment to induce acute inflammation of the colon and sensitize afferent pathways in the pelvis. Control groups were subjected to intracolonic saline treatment.

### TNBS-Induced Colonic Inflammation and Visceral Pain Model

Colonic inflammation was induced by administration of TNBS (12.5 mg/ml) in 50% ethanol solution. Rats were fasted for 24 h before the procedure to provide better access to the colonic lumen. Animals were briefly anesthetized with isoflurane (VEDCO Inc., St. Joseph, MO, USA), a 7–8 cm long catheter made of polyethylene tubing and attached to a 1 cc syringe was inserted into the rat colon for enema administration. After TNBS administration, an animal was held by the tail to avoid any spill of instilled liquid. The severity of experimental colitis was confirmed by the assessment of disease activity index, histological evaluation of the colonic wall upon tissue collection, and biochemical evaluation of myeloperoxidase level as previously published by our group (Pan et al., 2010; Asfaw et al., 2011; Lei and Malykhina, 2012). Control groups received intracolonic instillation of saline by enema. Since activation of transcription factors occurs quite rapidly after an intervention, we chose earlier time points to evaluate phosphorylation of Mecp2 and CREB in spinal and sensory neurons. Specifically, rats from TNBS-treatment group were sacrificed at 1 h, 2 h, 6 h, 24 h and 3 days after the treatment. Tissue samples from LS (L6-S2) spinal cord and L6-S2 DRGs were isolated from each animal and either snap-frozen in liquid nitrogen for molecular studies, or fixed for histological experiments.

### Protein Extraction and Western Blotting

Total protein was extracted from frozen spinal cord and DRG specimens by homogenizing the tissues in ice-cold lysis buffer containing 25% glycerol, 62.5 mM Tris-HCl, 1× protease inhibitors (Roche, Complete mini) and phosphatase inhibitors (Roche, PhosSTOP). Ten percentage SDS was added to the samples, vortexed and boiled for 4 min. The extracts were centrifuged at 10,000 rpm for 15 min at 4°C, and supernatants with the total protein were collected. Protein concentration in each sample was detected using BCA protein assay kit (Thermo Fisher Scientific, Rockford, IL, USA). Bovine serum albumin was used to generate the standard curve. Each protein sample was diluted 1:20 with 1% SDS. All standards and samples were run in duplicate. The absorbance was measured at 562 nm on the Synergy 2 Multi-Detection Microplate Reader (BioTek Instruments, Winooski, VT, USA) and data analysis was performed using Gen5 Microplate Data Collection and Analysis Software (BioTek Instruments, Winooski, VT, USA).

Extraction of nuclear protein was performed on spinal cord specimens only due to limited amount of DRG tissues and low total protein yield. Nuclear portion of the protein was extracted using nuclear extraction kit from Cayman (cat#10009277), collected by centrifugation and immunoprecipitated with either anti-Mecp2 rabbit polyclonal antibody (Dr. Zhou’s lab), or anti-CREB, rabbit monoclonal antibody (Cell Signaling, #9197) or normal rabbit IgG overnight at 4°C. Samples were analyzed by 4%–12% SDS-PAGE followed by Western blotting using anti-Mecp2, mouse monoclonal antibody (Abcam, #ab50005) and anti-CREB, mouse monoclonal antibody (Thermo Fisher, Waltham, MA, USA). For additional experiments, expression of phosphorylated-RAC-alpha serine/threonine-protein kinase (p-Akt) and p-extracellular signal-regulated kinases 1/2 (p-ERK1/2) was tested in nuclear extracts from the spinal cord using respective primary antibodies from Cell Signaling (Table 1). After wash with TBS-T wash buffer (TBS containing 0.1% Tween-20), the secondary antibody ECL Plex goat-anti-mouse IgG-Cy3 were used. The nitrocellulose membrane was read using Typhoon 8600 Imager (Amersham Biosciences).

**Table 1:** List of antibodies and their concentrations used in immunohistochemical (IHC) and Western blotting experiments.

	Supplier	Application and dilution
**Primary antibodies**		
Mecp2	Dr. Zhou’s lab	IHC: 1:1,000
Mecp2	Abcam, #ab50005	IHC: 1:5,000; WB:1:1,000
pS421Mecp2	Dr. Zhou’s lab	IHC: 1:2,000; WB:1:1,000
CREB	Cell Signaling, #9197	IHC: 1:1,600; WB: 1:1,000
pS133CREB	Cell Signaling, #9198	IHC: 1:1,000; WB: 1:1,000
p-Erk1/2	Cell Signaling, #4370	WB: 1:2,000
p-Akt	Cell Signaling, #4060	WB: 1:2,000
α-actin	SantaCruz, sc-17829	WB: 1:800
**Secondary antibodies**		
Alexa Fluor 555	Cell Signaling, #4413	IHC: 1:1,000
Alexa Fluor 488	Cell Signaling, #4408	IHC: 1:1,000
Rabbit IgG, Cy5	GE Healthcare, PA45011	WB: 1:2,500
Mouse IgG, Cy3	GE Healthcare, PA43009	WB: 1:2,500

### Immunohistochemical Labeling and Cell Counting

To evaluate intracellular localization of the transcription factors of interest, spinal cord and DRG sections underwent immunohistochemical (IHC) labeling. First, the animals were perfused through the ascending aorta with 100–150 ml saline followed by 300 ml 4% paraformaldehyde (PFA) in 0.1 M phosphate buffer (pH 7.4). L6-S2 spinal cord segments and respective DRGs were removed, placed in PFA overnight at 4°C, then cryoprotected in 30% sucrose for 48 h. Sections were then embedded in Tissue-Teck^®^ OCT compound, and sectioned on a cryotome at 8 μm increments. Frozen tissue sections were permeabilized with soak buffer, PBS containing 0.2% Triton X-100 for 10 min and rinse buffer, 10% methanol in PBS for 5 min. After blocked with blocking buffer, 3% normal goat serum in PBS for 1 h at R/T, tissue sections were incubated with primary antibodies, Mecp2 (rabbit antibody, 1:1,000, Zhou’s lab), pS421Mecp2-S421 (rabbit antibody, 1:2,000, Zhou’s lab), CREB (Cell Signaling, #9197, rabbit monoclonal antibody, 1:1,600) or pS133CREB (Cell Signaling, #9198, rabbit monoclonal antibody, 1:800) diluted with antibody dilution buffer, 1% normal goat serum in PBS containing 0.2% Triton X-100 at 4°C overnight. After washed with PBS, sections were incubated with rabbit Alex Fluor 555 Conjugated antibody (Cell Signaling, #4413), 1:1,000 at R/T for 1 h in dark and then stained with DRAQ5 (Cell Signaling, #4084), 1:1,000 dilution or DAPI (Cell Signaling #4083), 1 μg/ml in PBS for 5 min at R/T after washed with PBS (Table 1).

Sections were mounted in Prolong Gold Anti-fade Reagent (Invitrogen, #36930) after quick rinse with PBS. A negative control to confirm the specificity of immunostaining was prepared using non-immune rabbit and mice serum in place of the primary antibody. For double immunofluorescence, sections were incubated with a mixture of two primary antibodies followed by a mixture of Alexa 568- and Alexa 488-conjugated secondary antibodies. Four rats were used for each group for IHC with 12–15 sections made per tissue, and 4–5 sections used for data collection and analysis. Images were acquired using a Leica DM5500B fluorescent microscope with a Leica DFC360 FX digital camera. All images were acquired using identical settings for laser power, detector gain amplifier offset and pinhole diameter in each channel. Image processing was performed using ImageJ and Adobe Photoshop, including identical adjustments for brightness, contrast, and levels in individual color channels and merged images.

### Fast Blue Retro-Labeling of Bladder Projecting Neurons in LS DRGs

Animals were anesthetized with sodium pentobarbital (60 mg/kg) injected intraperitoneally and kept under anesthesia using 1.5% isoflurane. A midline laparotomy was performed to gain access to the urinary bladder. Fast blue (Polysciences Inc., Warrington, PA, USA; 1.5% w/v in water) was injected into the urinary bladder wall using a Hamilton syringe at 6–8 sites (Hamilton Company, Reno, NV, USA). Incisions were sutured in layers under sterile conditions and rats were allowed to recover on a warm blanket and monitored for signs of pain or discomfort. Buprenorphine (2.0 mg/kg) was injected subcutaneously for pain management.

### Statistical Analyses

Data are expressed as means ± SEM. Analysis of gene and protein expression was done as previously described in Pan et al. (2010). The differences between the groups were statistically analyzed using two-way repeated measures ANOVA followed by group comparison between the groups using Bonferroni’s *t*-test method (Systat Software Inc., San Jose, CA, USA). Fisher’s exact test was used to compare the number of neurons between the treatments. *p ≤ 0.05 was considered statistically significant.

## Results

### Intracolonic TNBS Treatment Triggers Rapid Mecp2 Phosphorylation in the Lumbosacral Spinal Cord

**Figure 1:**
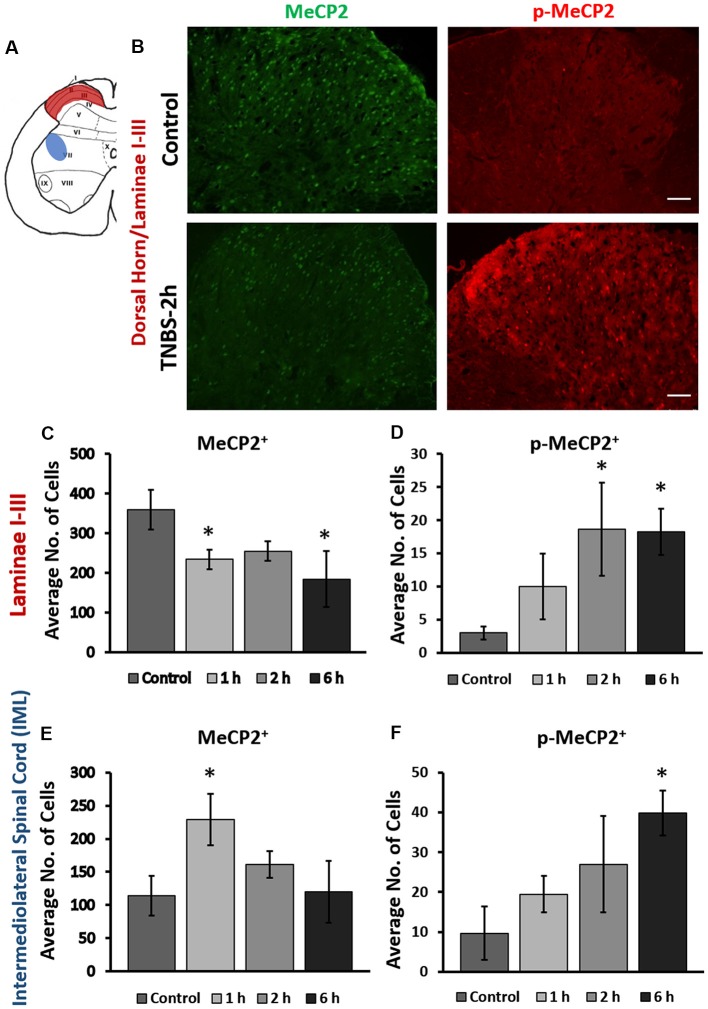
Intracolonic 2,4,6-trinitrobenzenesulfonic acid (TNBS) treatment led to rapid changes in cellular expression of methyl-CpG-binding protein 2 (Mecp2) and pS421Mecp2 in the spinal afferent and efferent region. **(A)** Schematic representation of rat lumbosacral (LS) spinal cord. Maroon region in the superficial dorsal horn (laminae I-III; afferent) and the blue region in the intermediolateral (IML) region (efferent) of the spinal cord indicate from where the data images were taken. **(B)** Representative immunohistochemical (IHC) images from the dorsal horn of the spinal cord from saline-treated and TNBS-treated rats at 2-h post instillation. TNBS instillation led to a decrease in the number of Mecp2-positive cells (left panels) and an increase in the number of pS421Mecp2-positive cells (right panels) compared to the saline-treated controls. Scale bars: 50 μm. **(C)** TNBS instillation led to a progressive decrease in the number of Mecp2-positive cells in laminae I-III (*n* = 16–20, *N* = 4 per group, *p ≤ 0.05 compared to control). **(D)** TNBS instillation led to a significant increase in the number of pS421Mecp2-positive cells in laminae I-III (*n* = 16–20, *N* = 4 per group, *p ≤ 0.05 compared to control). **(E)** TNBS instillation led to a transient increase and subsequently decrease in the number of Mecp2-positive cells in the IML region of the spinal cord at 1 h after instillation (*n* = 16–20, *N* = 4 per group, *p ≤ 0.05 compared to control). **(F)** TNBS instillation led to a progressive increase in the number of pS421Mecp2-positive cells in the IML region of the spinal cord (*n* = 16–20, *N* = 4 per group, *p ≤ 0.05 compared to control).

Expression of Mecp2 and pS421Mecp2 was evaluated at several time points (1 h, 2 h, 6 h, 24 h, and 3 days) in the superficial dorsal horn (laminae I-III, maroon region, Figure 1A) and intermediolateral (IML) portion (blue region, Figure 1A) of the LS (L6-S2) spinal cord of animals underwent intracolonic treatment of TNBS or saline (control groups). In control animals (2 h post saline instillation), Mecp2 expression was detected in the nuclei of majority of cells in the superficial dorsal horn (360 ± 45 cells per image, Figure 1B, upper left panel; Supplementary Figure S1), whereas very few cells (3 ± 1 cells) showed detectable level of pS421Mecp2 (Figure 1B, upper right panel). Intracolonic TNBS treatment led to a significant decrease in the number of Mecp2-positive cells (Figure 1B, bottom left panel; Figure 1C; 234 ± 34 cells per image at 1 h, and 184 ± 57 cells per image at 6 h; *p ≤ 0.05 compared to control) and a significant increase in the number of pS421Mecp2-positive cells (Figure 1B, bottom right panel, Figure 1D; 19 ± 10 cells per image at 2 h; *p ≤ 0.05 compared to control). The downregulation of Mecp2 expression and upregulation of pS421Mecp2 persisted until 6 h post-treatment (Figures 1C,D; *p ≤ 0.05 compared to control). These data suggest that peripheral inflammatory insult induced rapid phosphorylation of Mecp2 in the LS spinal cord.

Intracolonic TNBS treatment also induced changes in the expression of Mecp2 and pS421Mecp2 in the IML region of the LS spinal cord (Figures 1E,F). LS IML neurons send efferent projections to the pelvic viscera and regulate visceral motor responses. Interestingly, although TNBS treatment led to a significant increase in the number of pS421Mecp2 positive cells (Figure 1F; 9.7 ± 7.7 cells in the control group vs. 39.7 ± 5.6 at 6 h post-treatment, *p ≤ 0.05 compared to control), the number of Mecp2-positive cells transiently increased at 1 h post-treatment (Figure 1E; *p ≤ 0.05 to control). The number of Mecp2-positive cells quickly returned to the baseline levels similar to the control (Figure 1E) and did not substantially change within the next 6 h. The transient spike in Mecp2 cellular expression and delayed increase in pS421Mecp2 level in the LS IML suggest that this transcription factor is likely to have a major impact in afferent rather than efferent pathways.

### Phasic Changes of CREB and pS133CREB Expression Following TNBS Treatment in the Superficial Dorsal Horn

**Figure 2:**
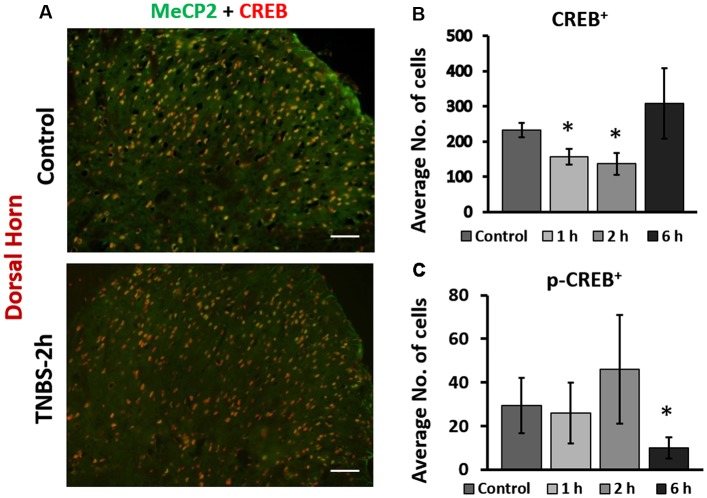
Intracolonic TNBS treatment led to rapid changes in nuclear expression of cAMP response element-binding protein (CREB) and pS133CREB in the dorsal horn of the spinal cord.** (A)** Representative IHC images from the dorsal horn of the spinal cord from control and TNBS-treated rats at 2-h post instillation. CREB-positive cells largely overlap with Mecp2 positive cells. TNBS instillation led to a decrease in the number of CREB-positive cells in the dorsal horn. Scale bars: 50 μm. **(B)** TNBS instillation led to a transient decrease in the number of CREB-positive cells in the dorsal horn of the spinal cord (*n* = 12, *N* = 4 per group, *p ≤ 0.05 compared to control). **(C)** TNBS instillation led to a significant decrease in the number of pS133CREB-positive cells in the dorsal horn of the spinal cord (*n* = 12, *N* = 4 per group, *p ≤ 0.05 to control).

We next examined the cellular expression of CREB and pS133CREB in the LS spinal cord following intracolonic TNBS treatment. IHC co-labeling of CREB and Mecp2 showed overlapping expression patterns in the superficial dorsal horn of LS spinal cord (Figure 2A). In control animals, the majority of Mecp2 positive cells (80 ± 6%) were also CREB positive (Figure 2A, upper right panel). TNBS treatment did not affect the overlapping expression pattern of Mecp2 and CREB in the same cells, suggesting possible co-regulation of Mecp2 and CREB in LS spinal neurons following colonic inflammation. However, inflammatory stimulation led to a significant and transient decrease in the number of CREB-positive cells in the LS superficial dorsal horn (control: 232 ± 27 cells per image; TNBS-1 h:157 ± 32 cells per image; TNBS-2 h: 137 ± 24 cells per image; Figures 2B,C, *p ≤ 0.05 compared to the control at both time points). The TNBS-induced decrease in the numbers of CREB positive cells recovered to the level of control group by 6 h post-instillation. TNBS treatment did not significantly increase the number of pS133CREB-positive cells at 2 h. However, the number of pS133CREB-positive cells was significantly decreased at 6 h following TNBS treatment (Figure 2C; control: 29 ± 12 cells per image; TNBS-6 h: 10 ± 3 cells per image, *p ≤ 0.05 compared to the control), followed by recovery to baseline levels at 3 days after the treatment (data not shown).

### Colonic Inflammation Induced Nuclear pS421Mecp2 and pS133CREB Expression in LS Spinal Cord

**Figure 3:**
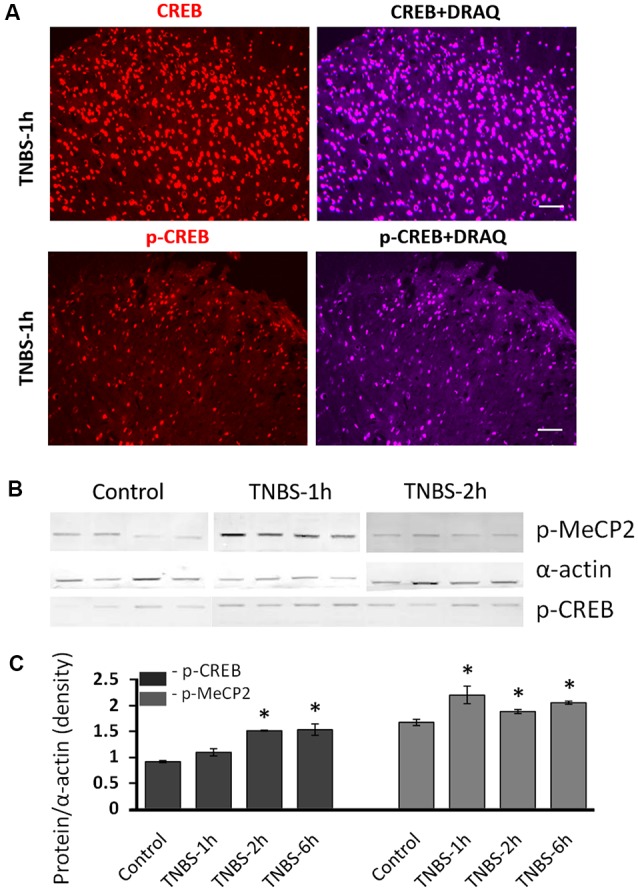
Molecular analysis of nuclear expression and phosphorylation of Mecp2 and CREB in the LS spinal cord following intracolonic TNBS treatment.** (A)** Representative images of CREB and pS133CREB expression (left panels) co-labeled with nuclear marker DRAQ (right panels) in the dorsal horn at 1 h after TNBS instillation. Both CREB and pS133CREB expression are restricted in the nuclei. Scale bars: 50 μm. **(B)** Representative Western blot experiment showing nuclear protein isolated from LS spinal cord of saline-treated control group and TNBS-treated group. There was no significant change of nuclear Mecp2 up to 6 h after TNBS instillation. **(C)** Nuclear pS421Mecp2 and pS133CREB were significantly upregulated in most of the time points after TNBS instillation (normalized to α-actin; *N* = 4 per group, *p ≤ 0.05 compared to control).

Our IHC data showed that the number of cells positive for Mecp2 and CREB changed following TNBS treatment, suggesting that expression levels of Mecp2, CREB and their phosphorylated isoforms were changed. To confirm TNBS-induced changes in protein expression of these transcription factors, we performed a quantitative analysis of nuclear protein extractions isolated from LS spinal cord from TNBS-treated and control rats. Co-labeling of spinal cord sections with nuclear marker DRAQ5 confirmed predominant nuclear location of CREB and pS133CREB in the cells of the superficial dorsal horn of the spinal cord (Figure 3A). Therefore, nuclear protein extractions from LS spinal cord were used in this experiment. Western Blot analysis of revealed a significant upregulation of pS421Mecp2 and pS133CREB within 6 h after TNBS treatment (Figure 3; *N* = 4 per group, *p ≤ 0.05 to control). We did not observe significant changes in the amount of nuclear Mecp2 at different time points.

### Protein Expression of Total and Phosphorylated Mecp2 and CREB Was Altered in LS DRGs Following Intracolonic TNBS Treatment

**Figure 4:**
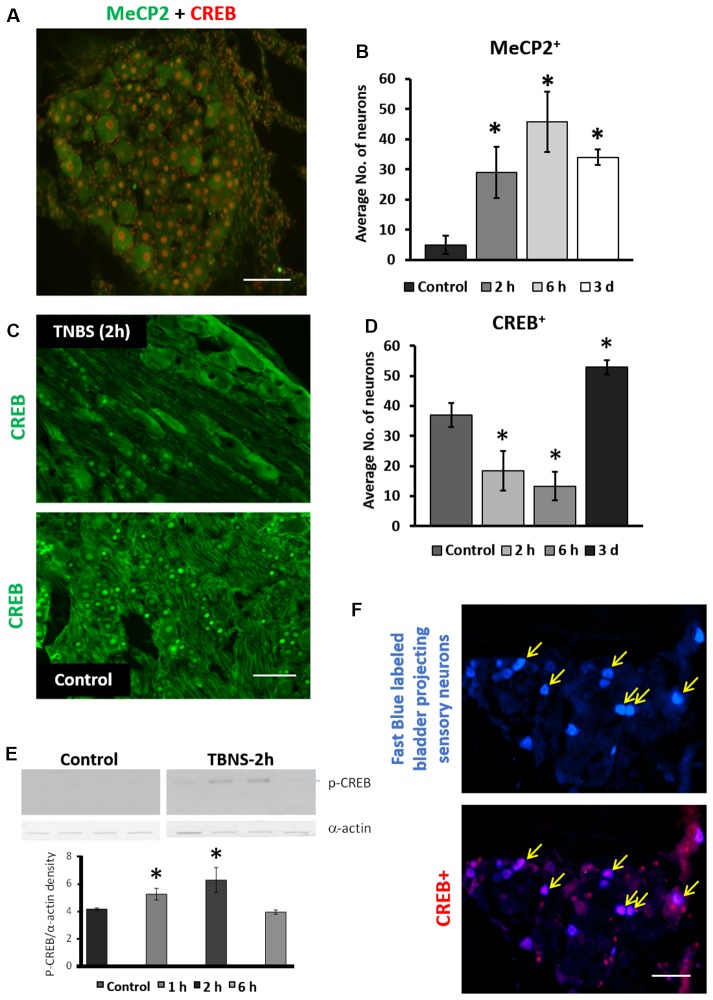
Intracolonic TNBS treatment led to phasic changes in neuronal expression of Mecp2, CREB and pS133CREB in LS sensory ganglia. **(A)** Representative image of IHC co-labeling of cellular expression of Mecp2 and CREB in LS dorsal root ganglias (DRGs; 2 h post-TNBS). Mecp2-positive labeling were detected in both the nuclei and cytosol in neurons as well as in satellite glial cells (SGCs), whereas CREB-positive labeling was mostly restricted to nuclei of the cells. Scale bar: 50 μm. **(B)** TNBS instillation led to significant increases in the number of Mecp2-positive neurons in LS DRGs up to 3 days post-TNBS treatment (*n* = 16, *N* = 4, *p ≤ 0.05 to compared to control). **(C)** Representative images of immunohistochemistry labeling of CREB in LS DRGs from control group and 2 h post-TNBS-instillation (TNBS-2 h). Scale bar: 50 μm. **(D)** TNBS instillation led to short-term decrease and long-term increase in the number of CREB-positive neurons in LS DRGs (*n* = 12, *N* = 4 per group, *p ≤ 0.05 compared to control). **(E)** Quantitative analysis of nuclear pS133CREB expression in LS DRGs of control group and TNBS-treated group (*N* = 4, *p ≤ 0.05 to compared to control). TNBS instillation led to short-term upregulation in nuclear pS133CREB in LS DRGs. **(F)** Representative images of nuclear CREB expression in bladder-projecting sensory neurons (yellow arrows) in LS DRGs. Bladder-projecting sensory neurons were retrogradely labeled with Fast Blue. CREB expression was detected in ~60% of bladder-projecting sensory neurons in LS DRGs (bottom panel). Scale bar: 50 μm.

To test the hypothesis that Mecp2 and CREB co-regulate neuronal gene expression in LS DRGs following TNBS treatment, IHC co-labeling of Mecp2 and CREB was performed in LS DRGs from both control and TNBS-treated animals (Figures 4A,C; Supplementary Figure S2). Mecp2 was present in both nuclei and cytosol of sensory neurons and satellite glial cells (SGCs) in the DRGs (Figure 4A; Supplementary Figure S2). Unlike Mecp2, CREB-positive labeling was predominantly nucleus-specific in both neurons and SGCs (Figure 4C). DRG neurons were identified based on their larger size and morphology, and the neurons positive for Mecp2 and CREB were counted in LS DRGs from both control and TNBS-treated animals (Figure 4A; Supplementary Figure S2). Colonic inflammation led to a significant increase in the number of Mecp2-positive neurons in LS DRGs up to 6 h post-treatment (Figure 4B, *N* = 3–4 sections per ganglion per animal; control: 5 ± 1 cells per image; TNBS-2 h: 29 ± 8 cells per image. TNBS-6 h: 45 ± 10 cells per image; *p ≤ 0.05 compared to the control at both time points). TNBS treatment also significantly decreased the number of CREB-positive neurons at the earlier time points (TNBS-2 h, Figure 4C) followed by a significant increase at 3 days post-treatment (Figure 4D; *n* = 16, *N* = 4 at TNBS-2 h and TNBS-6 h, *n* = 12, *N* = 4 at TNBS-3 days; *p ≤ 0.05 compared to control), a trend similar to those in the dorsal horn of the spinal cord.

To evaluate the nuclear expression and phosphorylation of Mecp2 and CREB in LS DRGs, nuclear extraction was prepared as described for the spinal cord. Western blotting analysis showed significant up-regulation in pS133CREB expression at 1 h and 2 h post-treatment (Figure 4E, *N* = 4, *p ≤ 0.05 to control). pS421Mecp2 expression using nuclear extracts from DRG samples did not reveal a detectable signal after running Western blotting. Overall, these data suggest rapid and transient changes in Mecp2 and CREB in sensory neurons of LS DRGs following TNBS treatment.

We previously reported that transient colonic inflammation induces pelvic pain and bladder overactivity due to visceral organ cross-sensitization occurring mainly *via* sensory pathways (Malykhina et al., 2006; Malykhina, 2007). To test whether TNBS affects expression of the transcription factors specifically in bladder-projecting sensory neurons, Fast Blue was injected into the bladder wall at several sites as previously described (Malykhina et al., 2006). Co-localization analysis showed that up to 60%–65% of bladder-projecting DRG neurons were positively labeled for CREB in both control and TNBS-treatment groups (Figure 4F).

### Inflammation of the Colon Led to Transient Phosphorylation of ERK1/2 and Akt in LS Spinal Cord and DRGs, Respectively

**Figure 5:**
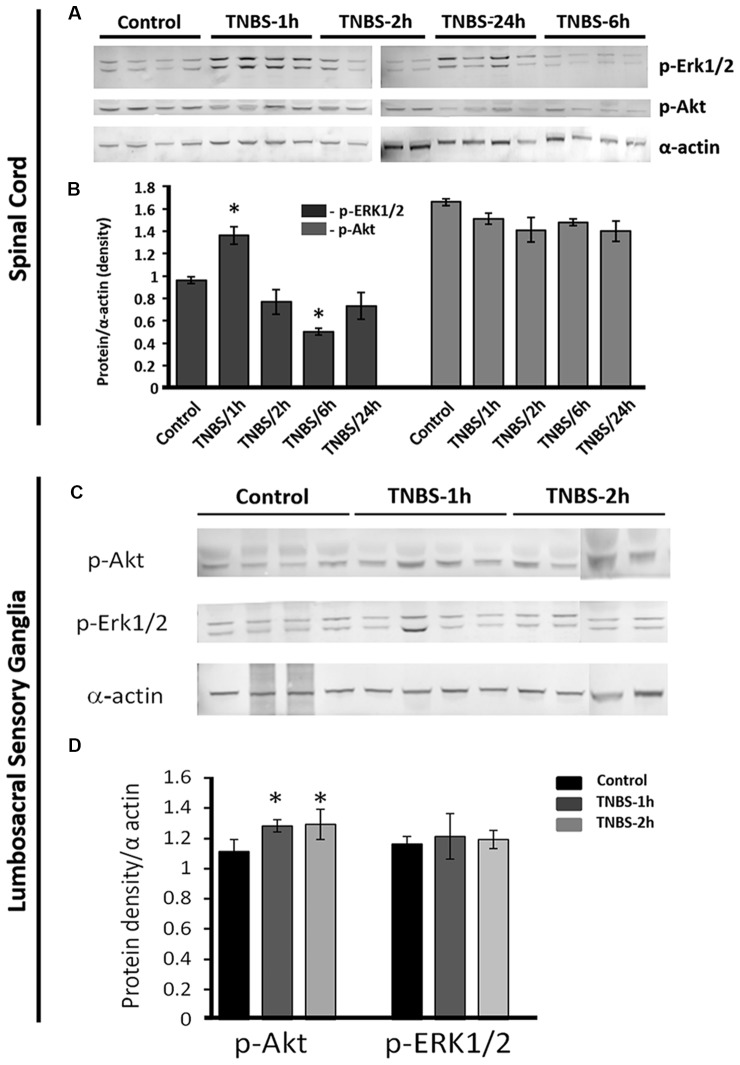
Intracolonic TNBS treatment triggers transient increases in nuclear phosphorylated-RAC-alpha serine/threonine-protein kinase (p-Akt) and p-extracellular signal-regulated kinases 1/2 (p-Erk1/2) in the LS spinal cord and nuclear p-Akt in LS-DRGs.** (A)** Representative Western blotting gels showing the nuclear content of p-Erk1/2 and p-Akt isolated from LS spinal cord from the controls and TNBS-treated groups at 1, 2, 6 and 24 h after TNBS instillation (*N* = 4 per group, *p ≤ 0.05 compared to control). **(B)** TNBS instillation induced changes in nuclear content of p-Erk1/2 and p-Akt (normalized to α-actin). Phosphorylation of ERK1/2 (p-ERK1/2) was significantly upregulated at 1 h post-TNBS treatment and down regulated at 6 h post-TNBS treatment in the LS spinal cords (*N* = 4 per group, *p ≤ 0.05 compared to control). **(C)** Representative Western Blotting experiment showing nuclear content of p-AKT and p-ERK1/2 isolated from LS DRG at 1 and 2 h post-TNBS instillation (*N* = 4 per group, *p ≤ 0.05 compared to control). **(D)** TNBS installation led to significant upregulation of nuclear p-Akt in LS DRGs at 1 h and 2 h post-TNBS treatment (*N* = 4 per group, *p ≤ 0.05 compared to control).

Recent studies revealed that Mecp2 regulates the signaling of Erk1/2 and Akt during neuronal differentiation (Mellios et al., 2018) and cancer cell proliferation (Zhao et al., 2017). Both Erk1/2 and Akt were also showed to play a modulatory role in central sensitization and nociceptive transmission (Chung et al., 2010; Nakanishi et al., 2010; Wang et al., 2010; Arms and Vizzard, 2011). Therefore, we tested if the changes in activation of Mecp2 occurred in parallel with alterations in nuclear ERK1/2 and Ark phosphorylation following TNBS treatment. Western blot analysis of nuclear extracts revealed that phosphorylation of ERK1/2 (p-ERK1/2) was significantly upregulated in LS spinal cord at 1 h post-TNBS treatment and downregulated at 6 h post TNBS-treatment (Figures 5A,B *N* = 4 per group, *p ≤ 0.05 compared to control). Phosphorylation of Ark (p-Akt) was also significantly elevated at 1 h and 2 h post-TNBS treatment (Figures 5C,D, *N* = 4 per group, *p ≤ 0.05 compared to control). No significant changes were detected in the expression levels of p-Akt or p-ERK1/2 in the LS DRGs.

## Discussion

In the present study, we observed early changes in cellular expression levels of Mecp2, CREB and their phosphorylated forms in the superficial dorsal horn and sensory ganglia following transient colonic inflammation, suggesting their potential roles in inflammation-induced visceral sensitization.

Alterations in expression and phosphorylation of Mecp2 have been previously reported in central and peripheral neural pathways in a large number of pain models (Tochiki et al., 2012; Ueda and Uchida, 2015; Zhang et al., 2015; Wang et al., 2016). For instance, in a rat model of spared nerve injury (SNI) -induced neuropathic pain, Mecp2 expression was decreased in the superficial dorsal horn at 7 days post-SNI surgery Complete Freund’s Adjuvant (CFA) injection (Tochiki et al., 2012). However, additional studies revealed an up-regulation of total Mecp2 expression in the dorsal horn of the spinal cord at 14 days after chronic constriction surgery (neuropathic pain model; Wang et al., 2016), and at 7 days after CFA injection in the ankle joint (inflammatory pain model; Tochiki et al., 2012). Interestingly, no significant changes in Mecp2 phosphorylation in the spinal cord at 7 days following CFA injection in the ankle joint nor after the SNI surgery were observed (Tochiki et al., 2012). Genome-wide microarray study using rat model of CFA-induced inflammatory pain found that in Mecp2 was phosphorylated in the superficial dorsal horn almost immediately after peripheral inflammation (Geranton et al., 2007). In addition, the expression of gene transcripts previously known to be repressed by Mecp2 showed upregulation within hours after inflammatory insult, but were mostly downregulated at 7 days after the intervention (Geranton et al., 2007). In our study, we focused on earlier time points after TNBS-induced colonic inflammation, and observed a decreased number of Mecp2-positive cells in LS spinal cord within the first 6 h after the treatment along with up-regulation in nuclear pS421Mecp2 content (at 2–6 h post-treatment). Similar elevation in p-Mecp2 was detected within early time frame (30 min to 1 h) in the Laminae I and II after CFA injection into the hind paw in rats (Geranton et al., 2008), suggesting that early phosphorylation of Mecp2 might be a common mechanism in inflammatory-induced pain models. Altogether, the published results and our data provide evidence that fluctuations in the expression and phosphorylation of Mecp2 in the superficial spinal cord depend on the pain model (neuropathic, inflammatory etc.), affected organ (somatic, visceral, etc.) and end time point after the insult.

Mecp2 may influence pain sensitivity *via* different mechanisms in post-mitotic neurons and during neurodevelopment (Leong et al., 2015; Lyst and Bird, 2015). Humans with mutations in *MECP2* gene and diagnosed with Rett syndrome show decreased pain responses and sensitivity (Downs et al., 2010; Peters et al., 2013). Recent studies using animal models of Rett syndrome established that Mecp2 knock-down confined to the sensory neurons in DRGs led to increases in axonal growth factors, abnormal axon sprouting, and resulted in altered pain sensitivity (Bhattacherjee et al., 2017). On the other side, overexpression of Mecp2 in mature spinal neurons in inflammatory pain models attenuated both acute mechanical and thermal pain (Zhang et al., 2015), an effect that might be due to upregulation of genes with analgesic properties.

### Potential Co-activation of Gene Transcription by Mecp2 and CREB

Mecp2 regulates the expression of transcription factor CREB1, which subsequently can enhance Mecp2 mediated gene activation (Chahrour et al., 2008). Therefore, we also investigated the expression of both CREB and pS133CREB in the superficial dorsal horn of the LS spinal cord after intracolonic TNBS treatment. The number of CREB-positive cells was transiently decreased within the first 2 h after the treatment followed by a decrease in the number of pS133CREB-positive cells. Prior experiments using mice that either lack or overexpress Mecp2 established that Mecp2 dysfunction induced changes in the expression levels of thousands of genes, but unexpectedly, the majority of genes (approximately 85%) were activated by Mecp2 (Chahrour et al., 2008). It was also shown that Mecp2 associates with the transcriptional activator CREB1 at the promoter region of an activated target gene but not a repressed target gene (Chahrour et al., 2008). The same group confirmed that Mecp2 directly binds to the promotor region of CREB1 based on the results of ChIP analysis and qRT-PCR data. Additional studies showed that the CREB-induced microRNA, miR132, represses Mecp2 translation (Klein et al., 2007). A article by Zhang et al. (2015) reported that time- and insult-dependent fluctuations in Mecp2 expression and phosphorylation may play an analgesic role in acute pain transmission *via* CREB/miR-132 pathway, which could be one of the protecting mechanisms during early response to noxious insult. A recent study by Bu et al. (2017) provided the first evidence that CREB signaling is involved in Mecp2 regulation in forebrain neurons differentiated from several human stem cell lines. The same group also established that pharmacological activation of CREB in female *Mecp2* heterozygous mice (*Mecp2^+/−^*) rescued several behavioral defects associated with down-regulation of Mecp2 expression.

Unlike in the spinal cord, analysis of LS DRG sections in our study revealed a significant up-regulation of total Mecp2 expression within hours following TNBS treatment. Differential spinal vs. ganglionic regulation of Mecp2 was previously observed in the model of neuropathic pain within days after SNI (Tochiki et al., 2012; Zhang et al., 2015). In the same model, Mecp2 expression was downregulated in DRGs from 2 days to 10 days (Zhang et al., 2015), but upregulated at 4 weeks post injury (Manners et al., 2016) confirming fluctuations in Mecp2 expression in DRGs in different pain states.

### A Potential Mechanism of BDNF Release in TNBS-Induced Pelvic Pain

Activation of peripheral transient receptor potential vanilloid 1 (TRPV1) receptors by painful stimuli is one of the established pathways triggering peripheral neuropeptide release (Pan et al., 2010). TRPV1 is a non-specific cation channel which opening depolarizes the cell membrane and, therefore, increases neuronal firing. Activation of TRPV1 channel causes an influx of calcium into the cell and can induce calcium-dependent phosphorylation and release of Mecp2 from the promoter region of affected genes, thereby, facilitating transcription (Chen et al., 2003). We previously found that TNBS-induced transient colonic inflammation triggers upregulation of CGRP, SP, and BDNF in sensory neurons in both spinal cord and DRGs, as well as an enhanced release of these neuropeptides in the urinary bladder (Pan et al., 2010; Lei and Malykhina, 2012). Therefore, it is possible that TRPV1-mediated rapid phosphorylation of Mecp2 occurs immediately after the peripheral insult, and, in turn, contributes to the induction of visceral pain. It was reported that *Mecp2*^+/−^ heterozygous mice had a decreased number of TRPV1-immunoreactive trigeminal ganglion neurons innervating the tongue (Suzuki et al., 2016). Following CFA injection in the tongue, the number of TRPV1- and Mecp2-expressing trigeminal neurons as well as Mecp2 protein expression in trigeminal ganglion were significantly increased in WT mice but not in *Mecp2*^+/−^ mice (Suzuki et al., 2016). These data further strengthened the previously suggested functional connection between TRPV1 activation and Mecp2 signaling in the induction of inflammatory pain.

### Erk1/2 and Ark as Early Co-activators in Inflammatory-Induced Pain Model

An up-regulation of pS421Mecp2 expression after inflammation observed in our study occurred in parallel with increased expression of p-ERK1/2 protein in the dorsal horn of the spinal cord and p-Akt in LS DRGs. ERK1/2 are known key players in signal transduction pathways involved in pain processing and peripheral sensitization (Wang and Zhang, 2012; Borges et al., 2015). Following noxious stimulation or inflammation, phosphorylated (activated) ERK1/2 (pERK1/2) expression was detected in primary afferents, spinal dorsal horn, and brain regions involved in pain processing (Dai et al., 2002; Ji et al., 2002; Obata et al., 2003; Carrasquillo and Gereau, 2007). However, recent studies determined that ERK2 alone can drive inflammatory pain but requires cooperation with ERK1 for neuronal survival (O’Brien et al., 2015). Concurrent phosphorylation of both Mecp2 and ERK1/2 detected in our experiments could suggest potential functional correlation between these signaling pathways, however, further studies are clearly warranted to address this suggestion.

### Study Limitations and Future Directions

We acknowledge several limitations of our study. First, we used only male rats in our experiments due to the nature of the utilized pain model (visceral pain), and to avoid well-known effects of estrous cycle hormonal fluctuations on pelvic pain development and persistence in females (Fillingim and Ness, 2000; Hassan et al., 2014). However, pelvic pain is more prevalent in females, therefore, future studies are warranted to assess the potential effects of ovarian hormones on Mecp2 and CREB expression and downstream signaling pathways. Second, while our data detected the changes in Mecp2 expression in the LS spinal cord and DRGs, we did not specifically determine in which cell types these changes occurred. Since the expression levels of Mecp2 were detected to be greater in neurons than in glia (Ballas et al., 2009; Jin et al., 2017), we assumed that the Mecp2 changes reflect more of neuronal expression levels. However, considering the importance of glia in the development of chronic pain states, the role of Mecp2 in spinal and sensory ganglionic glia during inflammatory pain requires further examination. Third, some of the differences in the results presented by us and other groups could be due to the use of different sources of total Mecp2 and pS421Mecp2 antibodies. We used Abcam mouse monoclonal [Mec-168] antibodies, and also Mecp2 and pS421Mecp2 antibodies generously donated to us by Dr. Zhaolan Zhou laboratory (University of Pennsylvania) while other groups used Sigma (Tochiki et al., 2012) and Millipore (Maezawa et al., 2009; Tochiki et al., 2012) anti-Mecp2 antibody. Future studies using molecular arrays will be very useful in determining how the changes in expression and phosphorylation of Mecp2 and/or CREB regulate the transcription of nociceptive genes in our model. Last, but not the least, our observations of early changes in expression of Mecp2, CREB, and their phosphorylation forms are descriptive in nature. Selective manipulation of Mecp2 and/or CREB signaling in spinal and sensory neurons are needed to determine precise functional role of these transcription factors in developing visceral hyperalgesia and chronic pain in inflammatory animal models.

## Author Contributions

AX, X-QP, and AM analyzed the data, performed experiments, conceived, discussed and outlined the experimental design. AX, X-QP, RM, and AM wrote the article, drafted and revised the article.

## Conflict of Interest Statement

The authors declare that the research was conducted in the absence of any commercial or financial relationships that could be construed as a potential conflict of interest.

## Abbreviations

TNBS: 2,4,6-Trinitrobenzenesulfonic acid
BDNF: Brain-derived neurotrophic factor
CGRP: Calcitonin gene-related peptide
CREB: cAMP response element-binding protein
CPP: Chronic pelvic pain
CFA: Complete Freund’s Adjuvant
DRGs: Dorsal root ganglias
Erk1/2: Extracellular signal–regulated kinases 1/2
IHC: Immunohistochemical
IML: Intermediolateral
LS: Lumbosacral
Mecp2: Methyl-CpG binding protein 2
Akt: RAC-alpha serine/threonine-protein kinase
SNI: Spared nerve injury
SP: Substance P
TRPV1: Transient receptor potential vanilloid 1 receptor.
