# Supplementary figures and images for: The Expression of Transcription Factors Mecp2 and CREB Is Modulated in Inflammatory Pelvic Pain
Source: Front Syst Neurosci. 2019 Jan 11;12:69. doi: 10.3389/fnsys.2018.00069 (PMC6336837; doi:10.3389/fnsys.2018.00069)

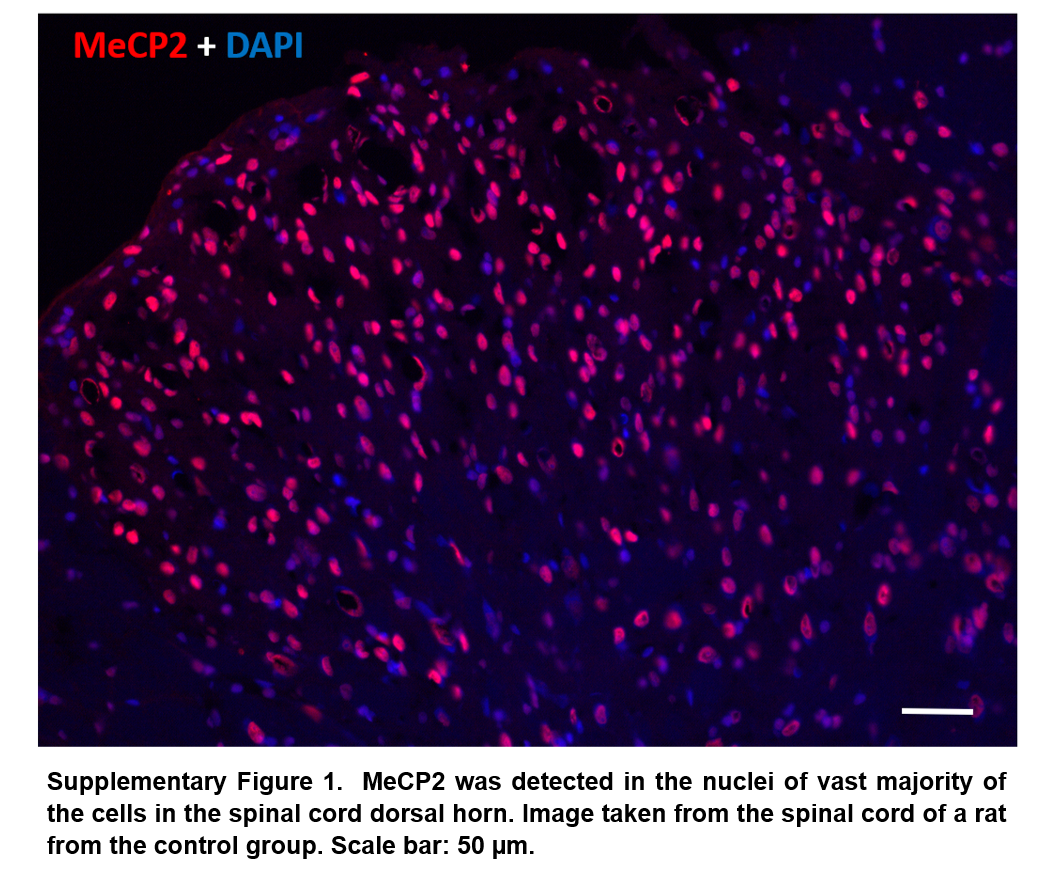

Supplement: Supplementary file 1 [file Image_1.tif]

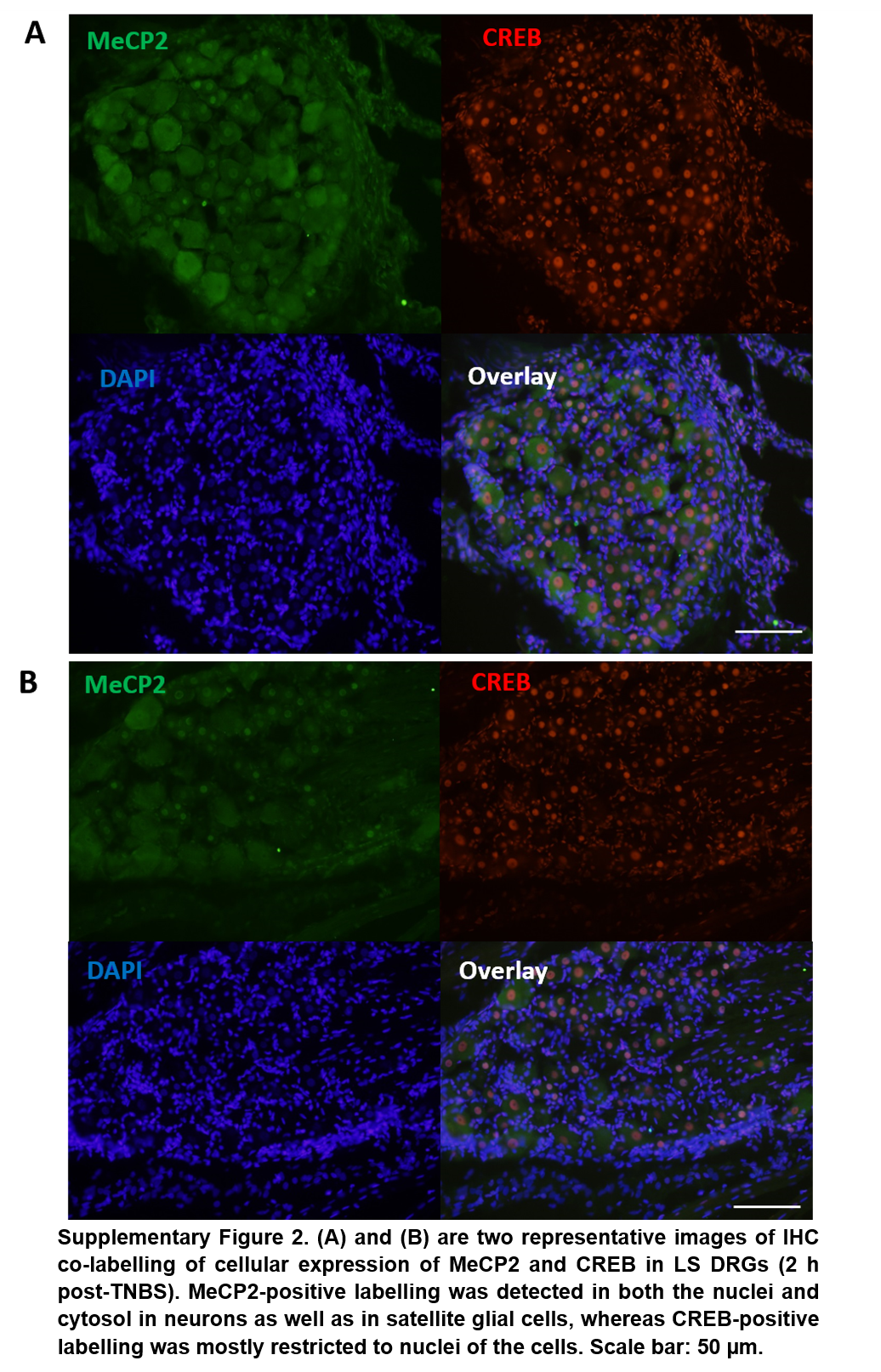

Supplement: Supplementary file 2 [file Image_2.tif]
